# Chromosome-level genome assembly of *Hippophae tibetana* provides insights into high-altitude adaptation and flavonoid biosynthesis

**DOI:** 10.1186/s12915-024-01875-4

**Published:** 2024-04-12

**Authors:** Guoyun Zhang, Yating Song, Ning Chen, Jihua Wei, Jianguo Zhang, Caiyun He

**Affiliations:** 1 State Key Laboratory of Tree Genetics and Breeding & Key Laboratory of Tree Breeding and Cultivation, National Forestry and Grassland Administration, Research Institute of Forestry, Chinese Academy of Forestry, Beijing, China; 2 Collaborative Innovation Center of Sustainable Forestry in Southern China, Nanjing Forestry University, Nanjing, China

**Keywords:** *Hippophae tibetana*, Chromosome-level genome, Adaptation, Flavonoid biosynthesis

## Abstract

**Background:**

As an endemic shrub of the Qinghai-Tibetan Plateau (QTP), the distribution of *Hippophae tibetana* Schlecht. ranges between 2800 and 5200 m above sea level. As the most basal branch of the *Hippophae* genus, *H. tibetana* has an extensive evolutionary history. The *H. tibetana* is a valuable tree for studying the ecological evolution of species under extreme conditions.

**Results:**

Here, we generated a high-quality chromosome-level genome of *H. tibetana*. The total size of the assembly genome is 917 Mb. The phylogenomic analysis of 1064 single-copy genes showed a divergence between 3.4 and 12.8 Mya for *H. tibetana*. Multiple gene families associated with DNA repair and disease resistance were significantly expanded in *H. tibetana*. We also identified many genes related to DNA repair with signs of positive selection. These results showed expansion and positive selection likely play important roles in *H. tibetana*’s adaptation to comprehensive extreme environments in the QTP. A comprehensive genomic and transcriptomic analysis identified 49 genes involved in the flavonoid biosynthesis pathway in *H. tibetana*. We generated transgenic sea buckthorn hairy root producing high levels of flavonoid.

**Conclusions:**

Taken together, this *H. tibetana* high-quality genome provides insights into the plant adaptation mechanisms of plant under extreme environments and lay foundation for the functional genomic research and molecular breeding of *H. tibetana*.

**Supplementary Information:**

The online version contains supplementary material available at 10.1186/s12915-024-01875-4.

## Background

Known as the Himalaya Plateau, the Qinghai-Tibet Plateau (QTP) is the world’s largest and highest plateau [1]. In the QTP, considerable geological uplifts have occurred seven times since the Pliocene [2]. For example, it has been estimated that the QTP mountains were uplifted three times between 0.6 and 1.3 million years ago (Mya) [3]. Increasing altitude has resulted in extensive glaciation on the QTP. Naynayxungla Glaciation, the largest glaciation in the QTP, began approximately 1.2 Mya and peaked between 0.6 and 0.8 Mya. The uplift of the Qinghai-Tibet Plateau (QTP) has led to significant climatic and environmental transformations in the plateau region. Low temperature, low oxygen, reduced pathogen incidence, and strong UV radiation now characterize the QTP conditions, creating an ideal environment for studying adaptive evolution [4, 5]. The diversifications of many endemic species are highly consistent with environmental changes and climate change [6]. A majority of genome-wide studies on adaptive evolution have been conducted on vertebrates and humans [7–9]. According to these studies, genes involved in hypoxia responses, energy metabolism, and skeletal development are undergoing positive selection and rapid evolution. Recently, a few studies have been conducted on wild plants in this region using whole-genome analysis to study adaptive evolution, including Tibetan hulless barley [10], wild barley [11], maca [12], and *Eutrema* [13].

As an original species on the QTP, *H. tibetana* is widely distributed in the Himalayas region [14]. In the *Elaeagnaceae* family, *Hippophae* is a small genus composed of five to seven species. Nitrogen-fixing is a function of all species of this genus [15]. The species of *H. tibetana*, which is morphologically uniform, lives in a variety of habitats ranging from 2800 to 5000 m in altitude [2]. *Hippophae* originated from ancient Mediterranean coast (the present Qinghai Tibet Plateau and Xinjiang) at 2500–4000 Mya. Phylogenetic analysis revealed that the divergence of *H. tibetana* from the other species of *Hippophae* genus occurred at around 3.15 million years ago [16]. The rapid rise of QTP may have altered the genetic factors of *H. tibetana*, reflecting their adaptation to the extreme environment.

Flavonoids from sea buckthorn (*H. rhamnoides* L.) were used to prevent and manage chronic diseases such as diabetes, cardiovascular disease, and cancer [17]. Sea buckthorn leaves, roots, stems, and fruits have high total concentration [18]. As a subgroup of flavonoids, flavonols are the most abundant in sea buckthorn. In sea buckthorn leaves and berries, the isorhamnetin glycosides are typically the most significant flavonols [19]. A variety of genetic and environmental factors influence flavonoid accumulation in sea buckthorn [18]. There are lots of genes involved in the flavonoid biosynthesis pathways, including phenylalanine ammonia lyase (*PAL*), cinnamic acid hydroxylase (*C4H*), 4-coumaric acid CoA ligase (*4CL*), chalcone synthase (*CHS*), chalcone isomerase (*CHI*), flavone synthase I and II (*FNSI* and *FNS II*), isoflavone synthase (*IFS*), flavanone-3-hydroxylase (*F3H*), flavonol 3′-hydroxylase (*F3′H*), flavonol 3′5′-hy-droxylase (*F3′5′H*), flavonol synthase (*FLS*), and dihydroflavonol 4-reductase (*DFR*). There is little information about the genes involved in flavonoid biosynthesis caused by historical genome duplication and gene family expansions and contractions. Genome assembly with annotations at the chromosome level could help identify key factors in flavonoid biosynthesis.

The draft genome of *H. rhamnoides* subsp. *mongolia* was assembled [20]. However, it has high heterozygosity (0.65%) and high rate of sequence repeats (> 65%). There were over 3000 fragmentary scaffolds in the genome of *H. rhamnoides* subsp. *mongolia*, which could easily have led to inaccuracies in genomic studies. In general, high-quality reference genomes for sea buckthorn species would be important for functional genomic studies.

Here, long-read sequencing technology, PacBio HIFI technology, and HiC sequencing technology were used to assemble a high-quality genome of *H. tibetana* with its high levels of repeat elements (> 65%). Using this newly obtained genome of *H. tibetana*, we performed comparative and evolutionary genomic analysis to clarify its origins and investigate evolutionary signals. In addition, by combining genomic and transcriptomic analysis, we identified key genes associated with the biosynthetic pathways of flavonoids in *H*. *tibetana*. We also confirmed the role of two genes (*HtC4H1* and *HtC4H2*) involved in the flavonoid biosynthetic pathway. The high-quality genome of *H. tibetana* helped us better understand how *H. tibetana* has adapted to the complex conditions on the QTP, and our results laid foundation resources for the molecular breeding of *H*. *tibetana* medicinal materials with higher flavonoid content.

## Results

### Chromosome-level reference genome of *H. tibetana*

**Fig. 1:**
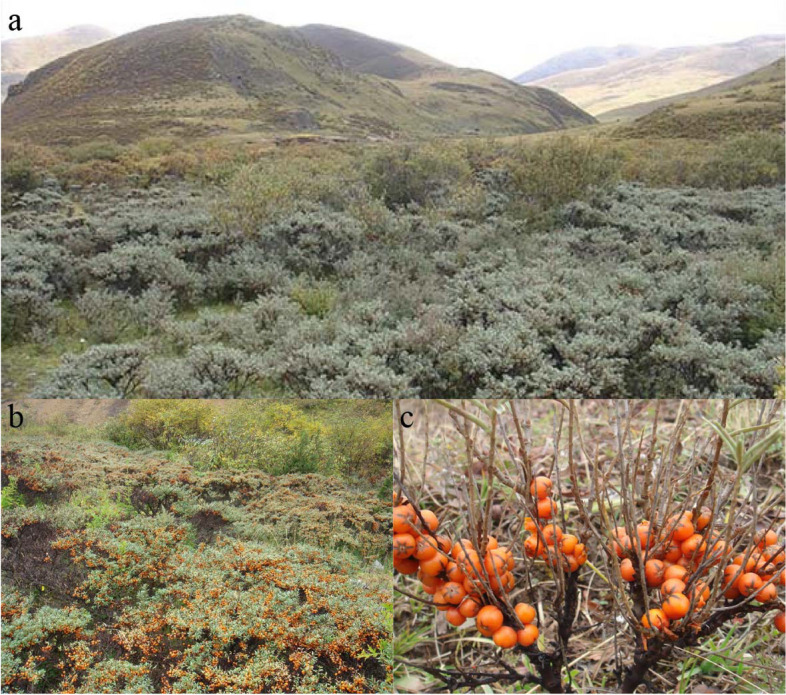
Sampling location and morphology of *H*. *tibetana*. **a** Sampling location of *H*. *tibetana*. **b** Photographs of *H*. *tibetana* trees. **c** Photographs of *H*. *tibetana* fruits

**Fig. 2:**
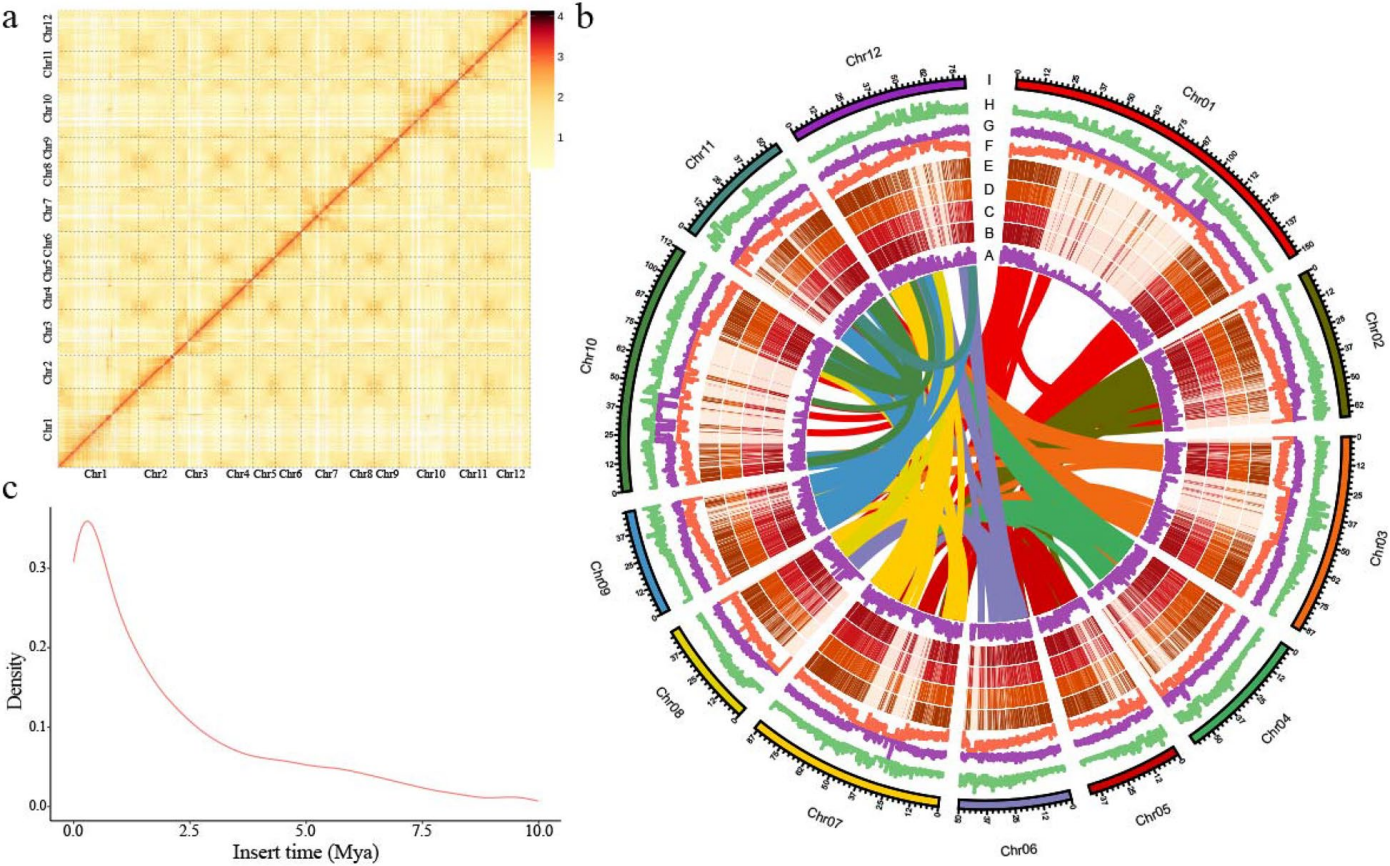
Overview of the *H*. *tibetana* genome. **a** Genome-wide all-by-all interactions among all sea buckthorn chromosomes. **b** (A) Gene density and distribution (non-overlapping window size, 100 kb); (B–E) gene expression levels (Log2 FPKM) in sea buckhorn stem, root, leaves, fruit; (F) density of repeats (non-overlapping window size, 100 kb); (G) density of Copia-type transposons (non-overlapping window size, 100 kb); (H) density of Gypsy-type transposons (non-overlapping window size, 100 kb); (I) sea buckthorn pseudo-chromosomes. **c** Insertion time distribution of LTR retrotransposons

Using the PacBio Sequel II platform, we generated ~ 50 × long reads to obtain a high-quality genome for *H. tibetana* (2*n* = 2*x* = 24) (Fig. 1). The estimated genome size was 997.42 Mb based on Illumina short-read sequencing data (Table 1, Additional file 1: Fig. S1). Assembling the dataset with Hifiasm yielded a 957.16-Mb assembly, which is 95.9% the estimated genome size. There were 90 contigs generated from the assembly, giving the genome assembly a contig N50 of 36.40 Mb. Then, the PacBio sequence assembly was polished with 52.08 Gb (~ 52 ×) of Illumina sequencing reads to remove sequencing errors found in single-molecule sequence datasets. After that, a total of 100.78 Gb of Hi-C data were generated, which was integrated into the assembly (Fig. 2a). Consequently, 12 pseudochromosomes (scaffold N50 size, 88.98 Mb) of *H*. *tibetana* genome were successfully assembled, which contains nearly 92.99% (890.09 Mb) of the assembled sequences (Fig. 2b, Additional file 2: Table S1). The assembled sequences showed low GC content of 29.8%. Compared with the genome sequences of *H. rhamnoides* subsp. *mongolia*, our assembly (contigs N50 of 36.40 Mb) was obviously improved, which contained 30 scaffolds. Genome assembly coverage was evaluated by mapping high-coverage Illumina sequencing reads against the assembled genome. There was a 99.91% mapping rate for Illumina DNA reads and 97.03% for Illumina RNA reads.

**Table 1:** Summary of the *H. tibetana* genomes

**Genomic feature**	***H. tibetana***	***H. rhamnoides* subsp. *mongolia***	***H. rhamnoides* subsp. *sinensis***
Total assembly size (Mb)	957.16	849.04	730.46
Contigs N50 (Mb)	36.40	2.15	2.82
No. of contigs	90	6357	2477
Scaffolds N50 (Mb)	88.98	69.52	65.06
No. of scaffolds	30	3642	1386
Percentage anchored to chromosomes (%)	92.99	89.42	96.67
GC content (%)	29.80	30.10	30.07
Number of genes	31,340	30,864	30,812
TE repeats (%)	65.77	67.81	47.48
Complete BUSCOs	98.20%	96.10%	98.40%

The assembled *H. tibetana* genome was 211 Mb larger than the reported genome of *H. rhamnoides* subsp. *sinensis*. In the *H. tibetana* genome, transposable elements (TEs) were more abundant than in the *H. rhamnoides* subsp. *sinensis* genome. It was found that TEs accounted for 605 Mb, or 66.01%, of the assembled *H. tibetana* genome, as compared to 335 Mb of TEs in the genome of *H. rhamnoides* subsp. *sinensis* (47.48%).

### Gene prediction and functional annotation

In *H. tibetana*, 31,340 protein-coding genes were annotated based on de novo, homologous, and RNA sequencing predictions. The average length of genes and coding sequences were 4387 bp and 1176 bp, respectively (Additional file 2: Table S2). According to mapping results, 96.8% (30,345) of the predicted genes have a corresponding function (Additional file 1: Fig. S2). In addition, the sea buckthorn genome was predicted to contain 391 miRNAs, 941 tRNAs, 3459 rRNAs, and 4425 snRNAs (Additional file 1: Fig. S3, Additional file 2: Table S3). We assessed the quality and completeness of this assembled genome using two strategies. First, the *H. tibetana* genome contained 237 (95.56%) core eukaryotic genes by a core eukaryotic gene mapping approach (CEGMA) analysis. Then, a 98.2% (1614 genes) BUSCO recovery score demonstrated the high completeness of the assembly of the *H. tibetana* genome (Additional file 2: Table S4). These parameters of the *H. tibetana* genome are better than other published *Elaeagnaceae Juss*. genomes.

Through multiple de novo prediction procedures, we identified and classified repeat sequences within the repeat library using RepeatMasker (Additional file 2: Table S5). Approximately, 65.77% of the *H*. *tibetana* sequences were identified as repetitive elements, including retrotransposons (51.11%), DNA transposons (3.3%), and unclassified elements (14.76%). The most common type of retrotransposons (50.48%) were long-terminal repeats (LTRs), of which Copia and Gypsy constituted 17.55% and 17.26% of the genome, respectively. In *H. tibetana*, retrotransposons might be associated with genome expansion. Based on *H. tibetana* genome sequences, we estimated the insertion dates of LTR retrotransposons to explore its evolutionary dynamics. In *H. tibetana*, LTR retrotransposons are most abundant at ~ 1.0 Mya (Fig. 2c). As a result of LTR retrotransposons’ proliferation, *H. tibetana* has probably evolved to become more diverse, speciated, and adapted to high altitude conditions.

### Evolution and gene family expansion/contraction analysis

**Fig. 3:**
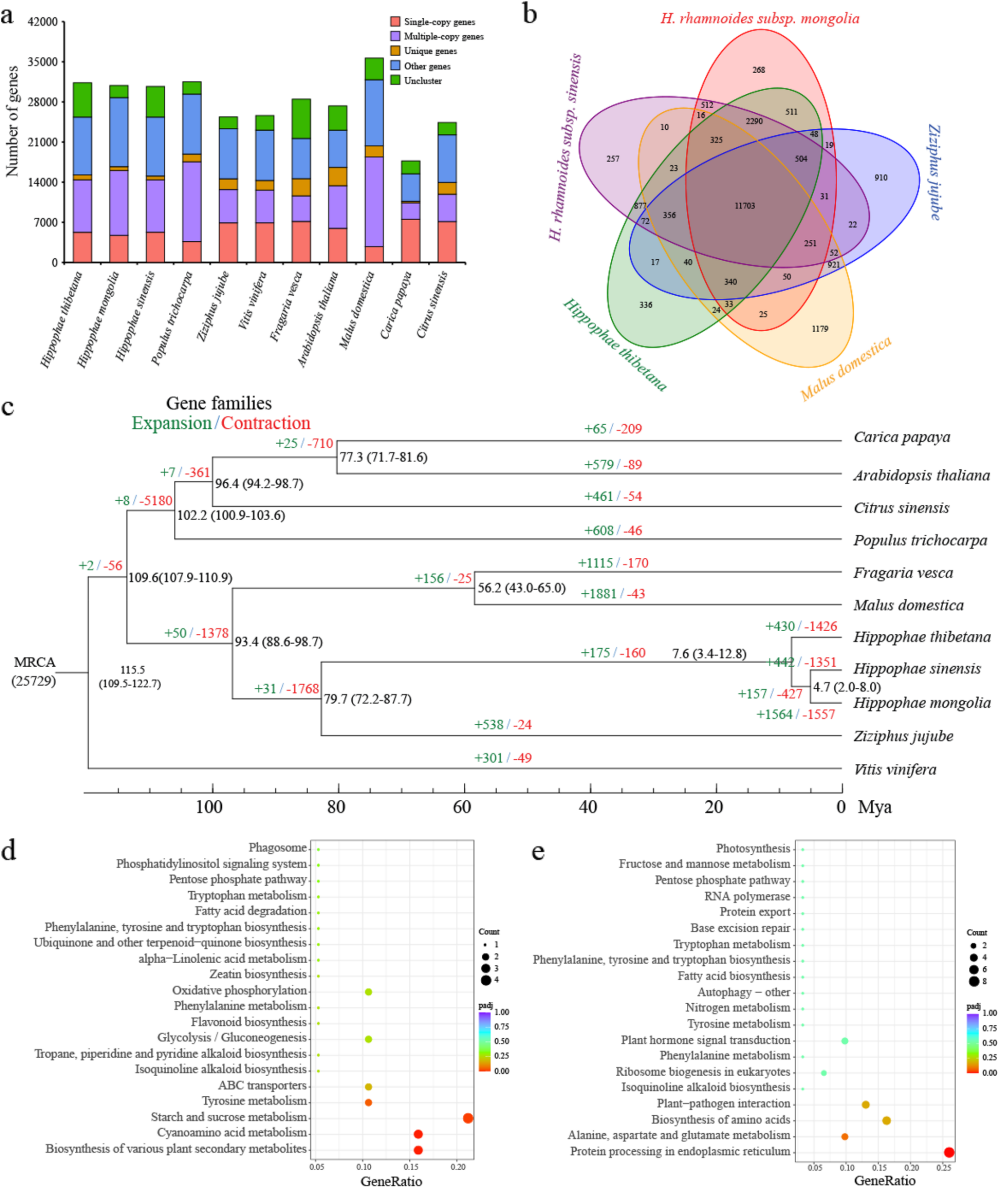
Characterization of *H*. *tibetana* genome evolution and gene family expansion. **a** Distribution of gene numbers and family sizes in *H. tibetana* and 10 other representative species. **b** Venn diagram showing unique and shared gene families between genomes of *H. tibetana* and 4 close relatives. **c** A species tree on the basis of 1064 single-copy orthologues from *H. tibetana* and 10 other representative species. The black numbers represent the divergence times from present. Green and red indicate the numbers of significantly (*p* < 0.05) expanded and contracted gene families. **d** KEGG functional classification of contracted gene families. **e** KEGG functional classification of expanded gene families

The genome sequences of *H. tibetana* and another 10 plants (*H. rhamnoides* subsp. *mongolia*, *H. rhamnoides* subsp. *sinensis*, *Populus trichocarpa*, *Ziziphus jujube*, *Vitis vinifera*, *Fragaria vesca*, *Arabidopsis thaliana*, *Malus domestica*, *Carica papaya*, and *Citrus sinensis*) were analyzed to determine the expansion and contraction of genes in the genome of *H*. *tibetana*. We totally identified 25,729 orthogroups from 11 plant genomes, 8602 of which were common between all 11 plants (Fig. 3a, b). *H. tibetana* genome contained 281 species-specific orthogroups, containing 857 genes. These 857 genes were found to be significantly enriched in a number of biological processes, including protein processing in the endoplasmic reticulum, plant-pathogen interaction, ribosome biogenesis in eukaryotes, and ubiquitin-mediated proteolysis (Additional file 1: Fig. S3).

The 1064 orthogroups with single-copy genes were identified from the 8602 common orthogroups. The single copy genes of the 11 plants were used to construct a phylogenetic tree (Fig. 3c). It is obvious that all 3 species of *Hippophae* L. (*H. tibetana*, *H. rhamnoides* subsp. *mongolia*, and *H. rhamnoides* subsp. *sinensis*) are clustered together. Also, in comparison with *H. tibetana*, *H. rhamnoides* subsp. *mongolia* and *H. rhamnoides* subsp. *sinensis* show the closest distance. According to the divergent analysis, *H. tibetana*, *H. rhamnoides* subsp. *mongolia*, and *H. rhamnoides* subsp. *sinensis* diverged from *Ziziphus jujube* at 79.7 Mya. The phylogenetic evolutionary relationship revealed that the *H. tibetana*, *H. rhamnoides* subsp. *mongolia*, and *H. rhamnoides* subsp. *sinensis* diverged at 7.6 Mya.

As a result of further analysis of gene clusters, numerous genes were found to have expanded or contracted in the 11 genomes. For the *H. tibetana*, a total of 430 expanded and 1426 contracted orthogroups were identified, respectively (Additional file 2: Table S6). Among 430 expanded orthogroups, 201 orthogroups with 1298 genes were significantly expanded (*p* < 0.01). According to the functional analysis of these 1298 genes (Fig. 3d), protein processing in the endoplasmic reticulum was the most enriched biological process. Among these expanded genes, a tryptophan decarboxylase (*TDC*) gene was found, which is involved in melatonin biosynthesis. Five CBL-interacting protein kinase (*CIPK*) genes were found, which play important roles in abiotic stress response. Two damage tolerance protein genes (*rad31-1* and *rad31-2*) were UBA-related genes required for DNA damage tolerance. Five genes involved in flavonoid biosynthesis were found. Three calcium-binding protein CML10 genes regulate cold tolerance. We identified a gene (Chr08.46) that is involved in photooxidative stress responses and cell death induced by UV-C. In addition, we also discovered genes associated with disease resistance in plants, including 4 pathogenesis-related gene 1 and 2 fatty acid 2-hydroxylase 1-like (*FAH2*, Chr09.2289, Chr11.212) genes, produce reactive oxygen species after chitin treatment. A total of 6 genes encoding alpha-glucan endo-1,3-beta-glucosidase have been found, which is considered as a defense factor against fungal pathogens. Five dirigent protein-like (DIR) genes were identified, which are involved in the plant disease-resistance response.

There is often an association between the adaptive divergence of closely related species and the contraction in the size of particular gene families. A total of 1426 gene families were identified as significantly contracted genes in the *H. tibetana* genome compared to the genomes of 10 species (*p* < 0.05) (Additional file 2: Table S6). Based on KEGG enrichment analysis results, contracted genes were significantly enriched in plant secondary metabolite biosynthesis, cyanoamino acid metabolism, and starch and sucrose metabolism (Fig. 3e). Among these contracted genes, 104 genes were annotated as resistance-related genes, including 8 *NBS-LRR* genes, 13 *LRR-RLK* genes, 45 *RLK* genes, 2 *RLP* genes, 6 *GLR* genes, 13 *GsSRK* genes, 7 *CRK* genes, 3 *GDSL* esterase/lipase genes, and 7 laccase genes.

**Fig. 4:**
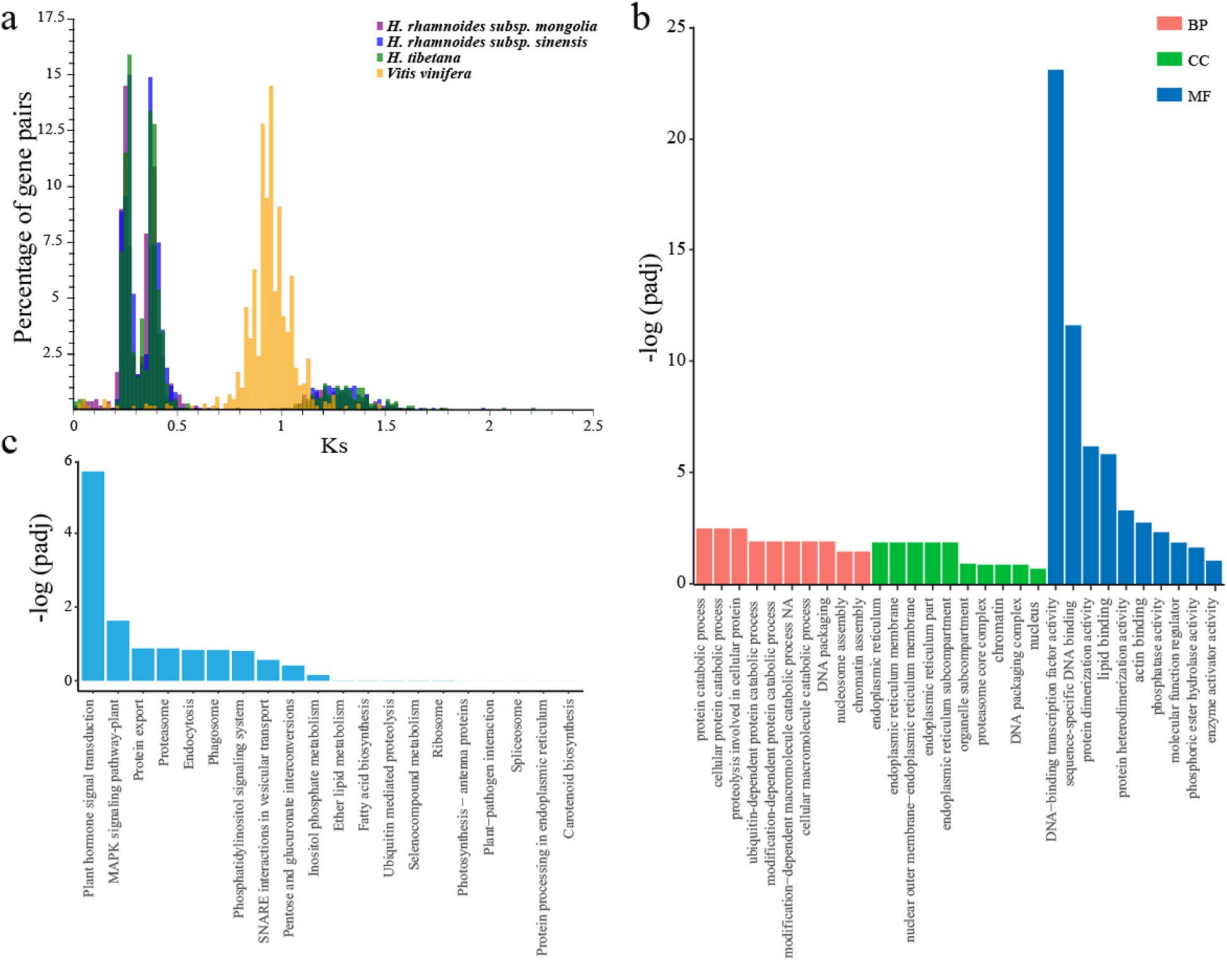
Identification of the WGDs in *H*. *tibetana* genome. **a** Ks distribution from orthologs between *H. tibetana* and other three species. **b** Gene Ontology (GO) functional classification of genes with WGDs. **c** KEGG functional classification of genes with WGDs

A whole-genome duplication (WGD) event is a key factor in the evolution of plants, animals, fungi, and other organisms. In order to investigate WGD in *H. tibetana*, we selected four species to perform a comparative genomics analysis. Based on the distributions of Ks of paralogous gene pairs in the *H. tibetana* genome, two recent clear peaks were found at 0.28 and 0.4 (Fig. 4a). There were two similar Ks peaks in the genomes of two closely related species, *E. mollis* and *H. rhamnoides*. Therefore, these two peaks might represent two closely WGD events in *H. tibetana*. These two WGD events were estimated to have occurred ~ 26 Mya and ~ 38 Mya. Phylogenetic evolutionary analysis indicated that these two WGD events occurred after *Hippophae* L. diverged from *Jujuba*.

As a driving force for plant evolution, WGD has the potential to endow genes with new functions. GO enrichment analysis of genes associated with WGD events showed that these genes were significantly enriched into protein catabolic process, cellular protein catabolic process, and proteolysis involved in cellular protein catabolic process (Fig. 4b, c). Our KEGG enrichment analysis found that genes associated with WGD significantly enriched into plant hormone signal transduction, MAPK signaling pathways, fatty acid biosynthesis, and flavonoid biosynthesis (*F3′H*, *CHS*, *C4H*, *CHI*, *DFR*) (Fig. 4c). Thus, WGD events may related with high level of fatty acid and flavonoid content in sea buckthorn.

### Candidate genes associated with high-altitude adaptation of the *H. tibetana*

Genes under positive selections were commonly associated with abiotic stresses, such as low temperature, low oxygen, reduced pathogen incidence, and strong UV radiation. The adaptive divergence of orthologs that show signs of positive selection usually occurs as a result of positive selection. Our study employed genomic sequences from *H. tibetana* and its 5 close species to conduct a positive selection analysis. We identified genes that show signs of positive selection from 1064 single-copy orthologs using the PAML 4 package’s branch-site model. The *H. tibetana* genome contained 212 genes possibly under positive selection (*ω* > 1, *p* < 0.05) (Additional file 2: Table S7). According to KEGG functional annotation, several of these genes are related to homologous recombination (RAD3-like DNA-binding helicase protein, DRD1 DEFECTIVE IN MERISTEM SILENCING 1, XRCC2 homolog of X-ray repair cross complementing 2, POLD3 DNA-directed DNA polymerase, RecQl3 DEAD/DEAH box RNA helicase family protein), Mismatch repair (5′-3′ exonuclease family protein, POLD3 DNA-directed DNA polymerase), ubiquinone and other terpenoid-quinone biosyntheses, base excision repair (poly ADP-ribose polymerase 3, POLD3 DNA-directed DNA polymerase), and plant-pathogen interaction (MKK6 MAP kinase kinase 6, calcium-binding EF-hand family protein). We identified genes that encode the RAD3-like DNA-binding helicase protein, DNA-directed DNA polymerase (POLD3), homolog of X-ray repair cross complementing 2 (XRCC2), DEAD/DEAH box RNA helicase family protein, 5′-3′ exonuclease family protein, and poly ADP-ribose polymerase 3, providing evidence that adaptive evolution in plants depends on positive selection of genes. In addition, two plant-pathogen interaction genes (MKK6 MAP kinase kinase 6 and calcium-binding EF-hand family protein) were also under positive selection, indicating that positive selection of genes may have a close correlation with plant-pathogen interaction in TQP.

### Genome comparison between *H. tibetana*, *H. rhamnoides subsp. mongolia*, and *H. rhamnoides subsp. sinensis*

**Fig. 5:**
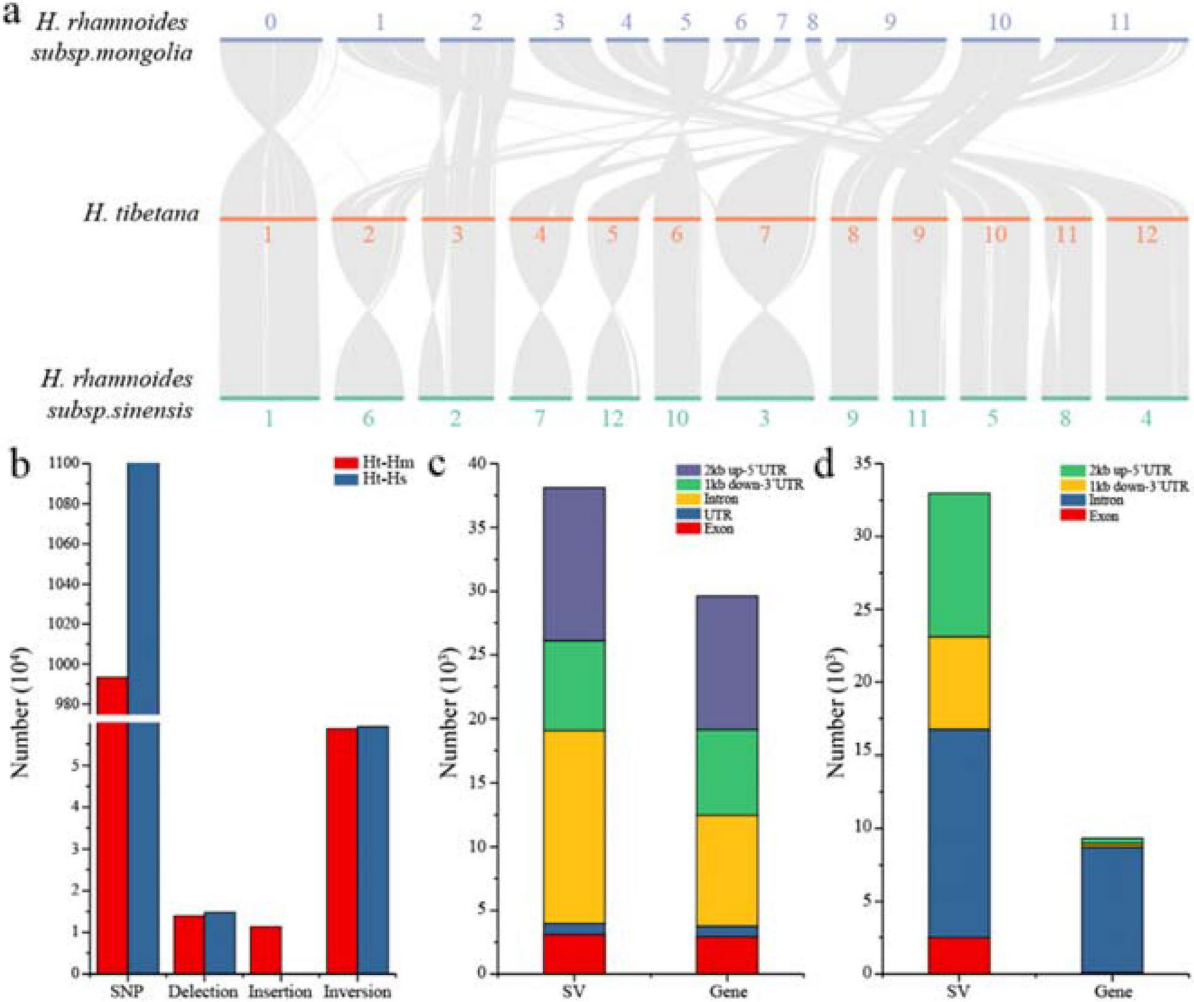
The comparison of *H. tibetana*, *H. rhamnoides* subsp. *mongolia*, and *H. rhamnoides* subsp. *sinensis* genomes. **a** Syntenic blocks share between the *H. tibetana*, *H. rhamnoides* subsp. *mongolia*, and *H. rhamnoides* subsp. *sinensis*. **b** The numbers of SNPs, delection, insertion, and inversion in the *H. tibetana* genome compared with *H. rhamnoides* subsp. *mongolia* and *H. rhamnoides* subsp. *sinensis*. genome. **c** The distribution of structural variation (SV) in the exon, intron, UTR, promoter (2 kb up-5′UTR), and downstream (1 kb down-3′UTR) regions of genes in the *H. tibetana* genome compared with *H. rhamnoides* subsp. *mongolia* genome. **d** The distribution of structural variation (SV) in the exon, intron, promoter (2 kb up-5′UTR), and downstream (1 kb down-3′UTR) regions of genes in the *H. tibetana* genome compared with *H. rhamnoides* subsp. *sinensis* genome

A variety of genetic variations, such as insertions and deletions, cause genetic diversity and phenotypic variation. Compared to *H. rhamnoides* subsp. *mongolia* and *H. rhamnoides* subsp. *sinensis*, *H. tibetana* exhibited obvious phenotypic differences, which allowed us to analyze the genomic variations between these three species. Full-genome alignment results revealed 237 syntenic blocks of *H. tibetana*, *H. rhamnoides* subsp. *mongolia*, and *H. rhamnoides* subsp. *sinensis*. These syntenic blocks contained 21,623 and 21,905 conserved genes covering 68.99% and 69.89% of the *H. tibetana* genome, respectively (Fig. 5a). In *H. tibetana*, *H. rhamnoides* subsp. *mongolia*, and *H. rhamnoides s*ubsp. *sinensis*, there are many collinear blocks, suggesting there is a high level of gene collinearity between the genomes (Fig. 5a). We identified 9,932,195 SNPs and 11,120,000 SNPs in *H. tibetana* genome compared with *H. rhamnoides* subsp. *mongolia* and *H. rhamnoides* subsp. *sinensis* genome (Fig. 5b, c). In addition, we identified 58,645 inversions, 13,967 deletion, and 11,305 insertion in *H. tibetana* genome compared with *H. rhamnoides* subsp. *mongolia* genome (Fig. 5b). Less deletion and insertion were found between *H. tibetana* and *H. rhamnoides* subsp. *sinensis* genome (Fig. 5b). Following this, we conducted structural variation analysis in the exon, promoter (2 kb up-5′UTR) and downstream (1 kb down-3′UTR) regions of gene in the *H. tibetana* genome. Compared with *H. rhamnoides* subsp. *mongolia* genome, we identified 2900 SV in the exon regions, 11,146 SV in the promoter regions, and 8616 SV in the downstream regions (Fig. 5b). Less SVs were found between *H. tibetana* and *H. rhamnoides* subsp. *sinensis* genome (Fig. 5c, d). *H. tibetana*, *H. rhamnoides* subsp. *mongolia*, and *H. rhamnoides* subsp. *sinensis* genomes show great variation possibly explaining their phenotypic differences.

### Investigation of genes involved in the flavonoid biosynthetic pathway

**Fig. 6:**
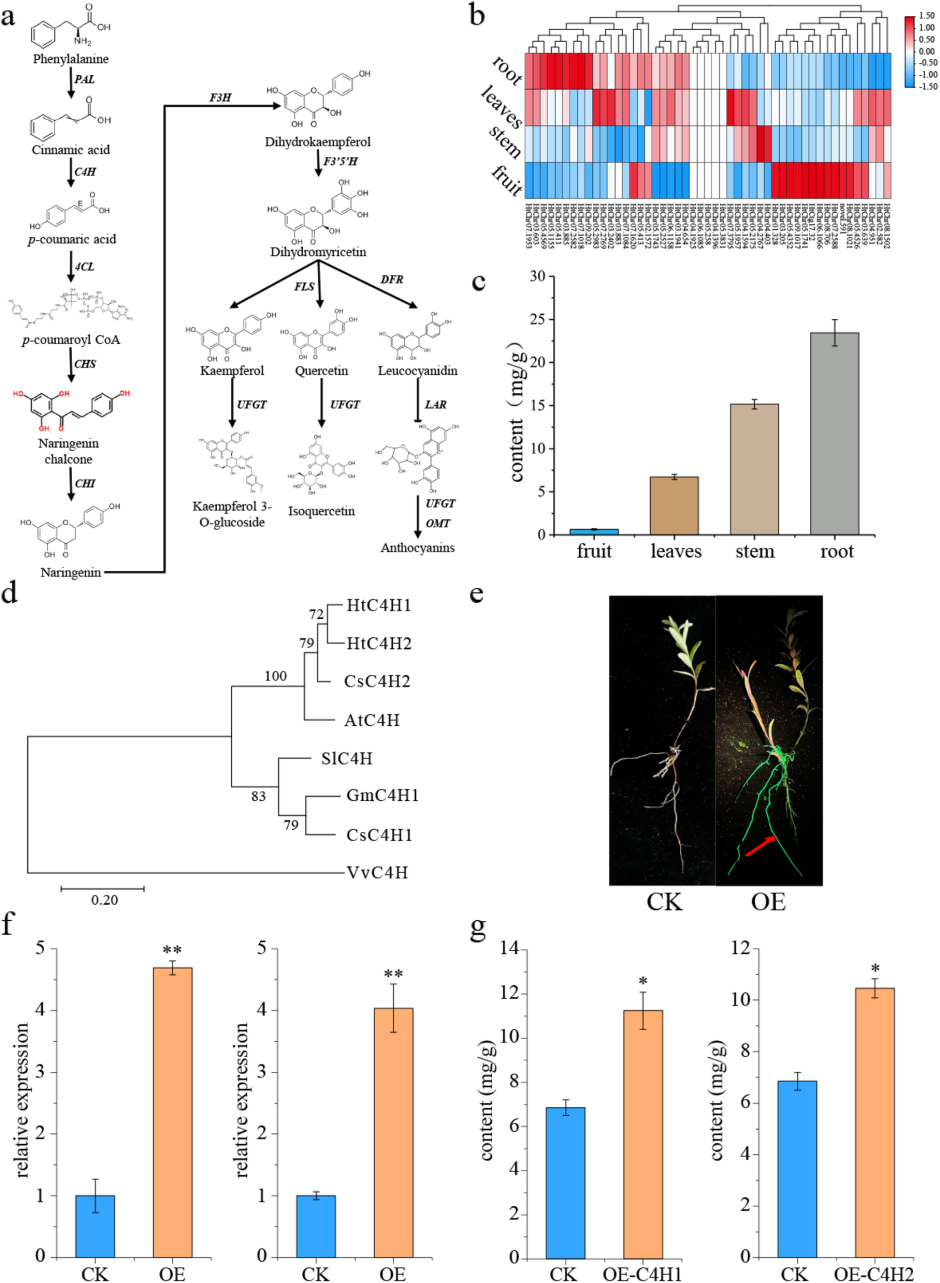
Genes associated with flavonoid biosynthesis in sea buckthorn. **a** Identification of structural flavonoid biosynthesis genes in *H. tibetana*. **b** Expression level of genes involved in the flavonoid biosynthesis pathway was detected in four different tissues of *H. tibetana*. The red colors indicated high expression levels. The blue colors indicated low expression levels. **c** the total flavonoid content of four different tissues of *H. tibetana*. **d** Maximum-likelihood trees of *C4H* family genes that were constructed using the amino acid sequences with 1000 bootstrap repeats. **e** Induction process of sea buckthorn hairy roots. CK, empty vector; OE-*HtC4H1*, overexpression lines (K599-1302-*HtC4H1*); OE-*HtC4H2*, overexpression lines (K599-1302-*HtC4H2*). **f** Relative expression levels of *HtC4H1* and *HtC4H2* in normal and transgenic hairy roots determined by qPCR. **p < 0.01, *p < 0.05. **g** Determination of flavonoids in CK and OE of hairy roots

Past researches reported the contents of phenylpropanoids and flavonoids were associated with the tolerance to UV light of Tibetan peach, *Arabidopsis*, and rice in TPQ [6, 21]. In plants, flavonoid biosynthetic genes have been identified and characterized (Fig. 6a). In the genome of *H. tibetana*, 49 homologs of genes associated with flavonoid biosynthesis were identified (Additional file 2: Table S8). Gene cluster analysis results showed that all these genes were divided into 3 groups (Fig. 6b). Most genes (44 genes) were expressed in all 4 different tissues, according to gene expression analysis. The root had higher expression levels for 17 genes involved in flavonoid biosynthesis than other tissues (Fig. 6b). In the fruit, 13 genes were more highly expressed in the flavonoid biosynthesis pathway (Fig. 6b). For example, the fruit exhibited extremely high expression levels of a F3H gene (HtChr08.706) (FPKM > 2571). In contrast, 2 *C4H* (HtChr04.654 and HtChr07.1953) genes have higher expression levels in the roots, leaves, and stem tissues, indicating the 2 genes might be related with the more flavonoid content in the roots, leaves, and stem of *H. tibetana* (Fig. 6c, d).

Genomics and RNA sequencing provide valuable information for metabolic engineering that can enhance flavonoid content (Additional file 1: Figs. S4 and S5). The phenolic biosynthesis pathway starts with *C4H*, which is important for flavonoid accumulation in many plants. The flavonoid content of *H. tibetana* hairy roots was increased by overexpressing two *C4H* genes (*HtC4H1* and *HtC4H2*) (Fig. 6e–g). We found that transgenic hairy roots showed higher flavonoid content compared with normal hairy roots (Fig. 6g). Hence, sea buckthorn flavonoids can be produced by cultivating hairy roots on a large scale.

## Discussion

Genomics sequences and annotations are fundamental to many genetic studies, including examining the inheritance of traits, comparing genetics, and studying evolution [22, 23]. Using PacBio, Hi-C, and Illumina data, we have assembled a high-quality chromosomal-level genome of *H. tibetana*, which is highly complete in protein-coding genes, repetitive sequences, and contigs. As a result, the genome sequences are excellent resources for studying a wide range of genetic studies in the *Hippophae* species and its related species. A larger genome size is found in *H. tibetana* than in other *Hippophae* species [20, 24]. Among *Hippophae* species, *H. tibetana*, *H. rhamnoides* subsp. *mongolia*, and *H. rhamnoides* subsp. *sinensis* experienced two very recent WGD events, which occurred at ~ 26 Mya and ~ 38 Mya. In close species, *Elaeagnus moorcroftii* and *Elaeagnus mollis* also have experienced two very recent WGD events [25]. All these results indicated that these two WGD events were species-special and may play important roles in *Elaeagnaceae*.

Past research revealed that significant expansion of LTR retrotransposons was found in the genome of several reported plants living in the Qinghai Tibet Plateau. For example, *C. himalaica* exhibits a dramatic increase in LTR retrotransposons following a dramatic elevational and climatic change during Miocene to Pliocene [26]. A higher proportion of retrotransposons were also found in the whole genome of Tibetan hulless barley [10], wild barley [11], maca [12], and *Eutrema heterophyllum* [13]. We also found that LTR retrotransposons have insertion at ~ 1.0 Mya in the *H. tibetana* genome, which is similar to these in *H. rhamnoides* subsp. *mongolia* [20]. The significant expansion of LTR retrotransposons may be related to the extreme environmental adaptability of plants in the QTP.

Diversification and adaptation of species are crucially influenced by gene family expansion and contraction. In Tibetan semi-wild wheat [27] and *C. himalaica* [26], genes involved in DNA repair and protein ubiquitination pathways were significantly expanded, including DNA excision repair protein ERCC-1, AP endonuclease 2 protein (APEX2), DNA polymerase delta subunit 2 (POLD2), DNA mismatch repair protein MLH1, and DNA repair and recombination protein RAD54. In the *Eutrema heterophyllum* genome [13], five expanded genes (*NBS1*, *RPA32*, *SNI1*, *KU80*, *XRCC4*) were found in the DNA repair pathway. In *H. tibetana*, five CBL-interacting protein kinase (*CIPK*) genes, two damage tolerance protein genes (*rad31*), three calcium-binding protein CML10 genes, and one phototropin-1 gene, which related with DNA repair, were also identified as expanded genes. In addition, a number of expanded genes were also found to be associated with disease resistance in plants, including four pathogenesis-related gene 1, two fatty acid 2-hydroxylase 1-like genes, six glucan endo-1,3-beta-glucosidase genes, and five dirigent protein-like genes. Overall, these results suggest that gene expansion is a key mechanism for plant adaptation to high altitudes. Otherwise, 8 NBS-LRR genes, 13 LRR-RLK genes, and 45 RLK genes were significantly contracted in *H. tibetana*. A similar observation was made about the genome of *C. himalaica*, where disease resistance R genes were also observed to show significant contraction [26]. In wheat, the *Pm21* gene encodes a CC-NBS-LRR protein that confers resistance to powdery mildew [28]. The *Arabidopsis LRR-RLK IOS1* plays a critical role in pattern-triggered immunity triggered by *BAK1* [29]. For rice, *Pi-d2* encodes a G-type RLK protein that provides resistance to *Magnaporthe grisea* [30]. Two *RLP* genes, which encode receptor-like proteins, are also involved in contracted genes. An important role played by *RLP1.1* is in responding to *Puccinia striiformis f.* sp. *Tritici* [31]*.* In addition, six GLR-contracted genes were also found in *H. tibetana*, which have a role in innate immune response. *Fov7*, encoding a GLR protein, confers resistance to *Fusarium oxysporum f.* sp. *vasinfectum* in upland cotton [32]. These genes may have evolved rapidly as a means of adapting to the rapidly changing pathogen spectrum of different species in QTP.

The adaptive divergence of orthologous genes has been accompanied by positive selection [26]. In the present study, an analysis of the *H. tibetana* genome totally identified 212 genes potentially under positive selection. A KEGG pathway enrichment analysis showed that these genes were associated with DNA repair, mismatch repair, base excision repair, and plant-pathogen interaction in the *H. tibetana* genome, which is similar with these in previous studies of *C. himalaica* [26]. For example, *CheY-like* genes demonstrated positive selection during adaptive evolution in *C. himalaica*. QTP is exposed to extremely intense UV radiation that could negatively impact plant growth and development, causing DNA, RNA, and protein damage [26]. A total of 7 genes under positive selection in the *H. tibetana* genome are related with the DNA repair pathway and homologous recombination pathway. It has also been found that two of them encode the DNA-binding helicase protein RAD3 and the DNA-directed polymerase protein POLD3, which was a component of DNA repair [26]. Furthermore, animals and plants are vulnerable to high UV-B radiation on the QTP. In Tibetan chicken, Tibetan hot-spring snake, and Tibetan barley, DNA repair pathways played an important role in adapting to high altitudes [7]. All these results indicated an important mechanism of genes under positive selection for high-attitude adaptive of *H. tibetana*.

In the *Hippophae* genus, flavonoids are a major subgroup of bioactive compounds that may play a key role in the prevention and management of chronic diseases such as diabetes and cardiovascular disease [17]. Flavonoids were found in sea buckthorn berries at a level of 1680–8570 mg/kg, more than in other plants with high flavonoids [18]. In many species, the genes and enzymes involved in flavonoid biosynthesis have been studied. Our study first identified and characterized 49 genes involved in the flavonoid biosynthesis pathway based on the genomic and transcriptomic analysis. Additionally, the WGD event can contribute to the formation and evolution of plant-specific pathways for metabolite biosynthesis in tea, yew, and *Scutellaria baicalensis* [22, 23]. In *H. tibetana*, we found five genes related to flavonoid biosynthesis undergone WGD, including *F3′H*, *CHS*, *C4H*, *CHI*, and *DFR*. Previous studies revealed the role of *F3′H*, *CHS*, *C4H*, *CHI*, and *DFR* in flavonoid biosynthesis in many plants [21]. A WGD event provided resources for the flavonoid biosynthetic pathway in *H. tibetana*.

The genomic and transcriptomic datasets of *H. tibetana* provided new insights and valuable tools into the flavonoid biosynthetic pathway. Recently, there have been no studies about manipulating flavonoid biosynthesis in *H. tibetana*. Flavonoid biosynthesis involves multiple enzymes, suggesting that metabolic flux may be limited by more than one enzyme. The *C4H* gene enhances the synthesis of flavonoids in *Nicotiana tabacum*, *Scutellaria baicalensis*, *Ginkgo*, and tartary buckwheat [33–36]. In this study, we first obtained transgenic *H. tibetana* hairy roots with significantly increased flavonoid content through overexpressing multiple genes (*HtC4H1* and *HtC4H2*) related to the flavonoid biosynthetic pathway. Consequently, the genes involved in flavonoid biosynthesis may be candidates for metabolically engineered flavonoid enhancement.

## Conclusions

This study generated one high-quality reference genome of *H. tibetana*, with a contig N50 of 36.4 Mb. The high-quality chromosome-level genome provides a reference for the understanding of high-altitude adaptation. In addition, transcriptomic and genomic investigations also provided an in-depth insight into flavonoid biosynthesis. Genomic data of *H. tibetana* will provide essential resources for further functional study with genome editing and improving *H. tibetana* as an economic crop for edible food and traditional Chinese medicine.

## Methods

### Plant materials, genomic DNA extraction, and sequencing

The *H. tibetana* plant was obtained from hongyuan county (32° 26′ 2.17″ N, 102° 22′ 11.61″ E), Sichuan Province in Eastern China. Following the manufacturer’s instructions, we extracted the genomic DNA from the young leaves of *H. tibetana* plants using a MiniBEST Plant Genomic DNA Extraction Kit (TaKaRa). The extracted cDNA was detected by a NanoDrop™ One UV-Vis spectrophotometer (Thermo Fisher Scientific, USA) and Qubit® 3.0 Fluorometer (Invitrogen, USA). After that, we used the BluePippin system (Sage Science, USA) to select and collect long DNA fragments. A 20-kb library was constructed according to the manufacturer’s instructions. This library was sequenced using the PacBio Sequel II system. We constructed Hi-C libraries according to the methods in past researches [37–39]. A formaldehyde solution was used to fix the leaf samples. Cross-linked DNA was digested with 400 U of DpnII restriction enzyme at 37 °C. T4 DNA ligase (NEB) was used for DNA ligation for DNA with biotin labeled. After reverse crosslinking, the DNA fragments were purified and fragmented to 300–500 bp. We used Dynabeads® M-280 Streptavidin (Life Technologies) to separate biotin-labeled DNA fragments. Hi-C libraries were constructed and sequenced on an Illumina HiSeq X Ten sequencer. According to the description in the past research, DNA libraries were constructed for Illumina sequencing [20]. Briefly, DNA segmentation, repairing DNA ends, and adding poly (A) adaptors were performed. Final DNA libraries were sequenced on the Illumina HiSeq 2500 system.

### Genome assembly and quality assessment

Illumina short reads were used for evaluating the genome size, heterozygosity ratio, and repeat sequence ratio using *k*-mer distribution analysis (*k* = 17). The CCS software is used to process the sequencing data of the Pacbio CCS platform to generate high-precision HiFi data. Based on PacBio reads, we assembled the contig-level genome using Hifiasm (parameters “-l 2 -k 51 -w 51 --write-paf --write-ec --h1 --h2”) [40]. Hi-C sequencing data were mapped to contigs sequences using the hiccup software to evaluate the quality of HiC data [41]. The ALLHiC (v0.9.8) software was used to assemble the chromosome-level genome using Hi-C data [42]. Fine adjustment of orientation results with the JuiceBox software [43]. We assessed the completeness of the assembled chromosome-level genome using conserved plant genes from the BUSCO (v3.0.2) and CEGMA (v2.5) databases. In order to assess coverage rate and average depth, BWA (v0.7.8) was used to map Illumina short reads to the assembled genome [44].

### Genome annotations

To annotate repetitive sequences in the assembled *H. tibetana* genome, we used RepeatModeler (v.1.0.5), RepeatScout (v.1.0.5), and LTR_FINDER (v.1.07) to build a de novo repeat library. Then, the repeat library (Repbase v15.02) was used to identify homologous repeat by RepeatMasker (v.4.0.7) and RepeatProteinMask (v.4.0.7) [45]. Tandem Repeats Finder (v4.09) was used to identify the tandem repeats in the *H. tibetana* genome [46].

We used 5 ab initio gene prediction programs for de novo prediction, including Augustus (v3.2.3), Geneid (v1.4), Genescan (v1.0), GlimmerHMM (v3.0.2), and SNAP (v2013.11.29). tBlastn (v2.2.26, *E*-value < 10e − 5) was used to predict gene models by aligning the protein sequences of five species (*Arabidopsis thaliana*, *Elaeagnus angustifolia*, *Fragaria vesca*, *H. rhamnoides*, *Malus domestica*) against the *H. tibetana* genome. RNA sequencing data from four different tissues were mapped to the *H. tibetana* genome for gene prediction using Tophat [47]. We conducted a comparative analysis of genes against the SwissProt, TrEMBL, and non-redundant protein databases using BLASTP, with a significance threshold set at an *E*-value of ≤ 1E − 05. The best hits were used to annotate the gene function. The GO and KEGG annotations for each gene were obtained by comparison with homologs in the GO and KEGG databases. The tRNAscan-SE (v1.4) and INFERNAL (v1.1.2) software were used to predict noncoding RNAs [48, 49].

### Comparative genomics and genome evolution analysis

Protein sequences from the ten genomes (*H. rhamnoides* subsp. *mongolia*, *H. rhamnoides* subsp. *sinensis*, *Populus trichocarpa*, *Ziziphus jujube*, *Vitis vinifera*, *Fragaria vesca*, *Arabidopsis thaliana*, *Malus domestica*, *Carica papaya*, *Citrus sinensis*) were downloaded from Ensembl Plants (http://plants.ensembl.org/index.html). Orthologous gene families were obtained using OrthoMCL (v1.4) [50]. MUSCLE (v3.8.31) was used to obtain a super-alignment matrix by aligning single-copy gene families. Then, an ML phylogenetic tree was constructed using MEGA7 [51]. Based on the TimeTree database (http://www.timetree.org/), we used MCMCTree to calculate the divergence time. The CAFE (v4.2) software was used to detect expanded and contracted gene families [52]. *H. tibetana* genomes were searched with LTR_FINDER to identify intact LTR retrotransposons [53]. The insertion times of LTR retrotransposons were calculated using LTR_retriever [54].

### Analysis of WGD events

In order to study the evolution of the *H. tibetana* genome, we compared the protein sequences from *H. tibetana*, *H. rhamnoides* subsp. *mongolia*, and *H. rhamnoides* subsp. *sinensis* and grape using BLASTP (*p*-value ≤ 1E − 07). MCScanX was used to detect syntenic blocks between genomes [55]. The synonymous substitution rate (Ks) values of gene pairs across syntenic blocks were calculated using KaKs_Calculator 2.0 [56]. GO and KEGG enrichment analyses were performed using clusterProfiler (v. 3.14.0) [57].

### Comparative analysis of *H. tibetana*, *H. rhamnoides subsp. mongolia* and *H. rhamnoides subsp. sinensis* genome

Using the MUMmer (v 3.23) software, we detected collinearity between *H. tibetana*, *H. rhamnoides* subsp. *mongolia*, and *H. rhamnoides* subsp. *sinensis* genomes [58]. An analysis of SNPs and indels was conducted with MUMmer (v 3.23) to compare *H. tibetana*, *H. rhamnoides* subsp. *mongolia*, and *H. rhamnoides* subsp. *sinensis* genome. The first step of this alignment was to use the parameter “-maxmatch -c 100 -l50” to align *H. tibetana*, *H. rhamnoides* subsp. *mongolia*, and *H. rhamnoides* subsp. *sinensis*. Then, “delta-filter -1” parameter was used to filter the alignment results. As described in previous studies, MUMmer (v 3.23a) was used to examine SV between *H. tibetana*, *H. rhamnoides* subsp. *mongolia*, and *H. rhamnoides* subsp. *Sinensis* [59]. As described by Zhang et al., SV was detected in the exons, promoters, and downstream regions of the *H. tibetana* genome [60].

### RNA extraction, sequencing, and analysis

Samples of root, stem, leave, and fruit tissues from three individuals were collected for total RNA extraction. We extracted total RNA from each sample using the MiniBEST Plant RNA Extraction Kit (TaKaRa, China) following the manufacturer’s protocol. The cDNA was synthesized using RNA to cDNA EcoDry Premix (Clontech) with oligo (dT) primer. The paired-end library was constructed based on the Paired-End sample Preparation kit protocol (Illumina, USA). RNA sequencing was performed on the NextSeq 500 platform (Illumina, USA). For RNA-seq analysis, we first used the HISAT2 software package to map RNA-seq data of roots, stems, leaves, and fruits to the *H. tibetana* genome [61]. The RSEM software was used to calculate the gene expression level as fragments per kilobase per million (FPKM) [62]. We used DESeq2 to identify differential expression genes with an adjusted *p*-value ≤ 0.05.

### Identification of genes involved in the flavonoid pathway

In order to identify the candidate genes related to the flavonoid biosynthesis in the *H. tibetana* genome, we mapped the genes involved in the flavonoid biosynthesis pathway in *A. thaliana* to *H. tibetana* genome using blastp. Genes with *E*-value < 1E − 5 were identified as candidate genes related to flavonoid biosynthesis. The MEGA 7 software was used to construct ML phylogenetic trees using the homologous protein sequences from the four species [51].

### Construction of transforming vectors and transformation of sea buckthorn

Gene-specific primers were used to clone the full-length open reading frames (ORFs) of *HtC4H1* and *HtC4H2* from young leaf cDNA of *H. tibetana*. Primers for the *HtC4H1* and *HtC4H2* genes containing Kpn I and Sal I cleavage sites were designed, and the *HtC4H1* and *HtC4H2* fragment was cloned from the extracted plasmid as the target fragment. The target fragment was inserted into the pCAMBIA1302-EGFP + 2 × P35s vector using a Seamless Cloning Kit (Beyotime, China) and transformed into an agrobacterium K599 competent cell (Huayueyang, China). Positive monoclonal colonies were selected for PCR verification and incubated with a TY liquid medium. When the OD600 value was about 0.4–0.6, the bacterial solution was injected into the stem base of sea buckthorn seedlings as described previously [63]. After growing in soil for 2 months, the roots with green fluorescence in sea buckthorn seedlings were collected.

### Determination of flavonoid content of transgenic hairy roots and qRT-PCR analysis

The Plant Flavonoid Content Assay Kit (Solarbio, China) was used to determine flavonoid content in transgenic hairy roots. A UV spectrophotometer was used to measure the absorbance of samples at 420 nm. As described above, the RNA was extracted from approximately 0.1 g of transgenic hairy roots and analyzed quantitatively using real-time PCR. Total RNA from transgenic hairy roots and normal hairy roots was extracted using the MiniBEST Plant RNA Extraction Kit (TaKaRa). A PrimeScript™ RT reagent Kit (TaKaRa, Dalian, China) was used to synthesize cDNA. With gene-specific primers (Table S8), we performed quantitative real-time RT-PCR (qRT-PCR) with TB Green® Fast qPCR Mix (TaKaRa, Dalian, China) on the ABI StepOnePlus Real-Time PCR System (Applied Biosystems). The conditions for thermal cycling were 50 °C for 2 min and 95 °C for 20 s and run at 95 °C for 5 s and 60 °C for 20 s for 40 cycles [33]. Relative mRNA levels were calculated using the 2^−ΔΔCt^ method. The sea buckthorn 18S gene was used to normalize the relative expression of genes. In all experiments, three biological replicates were examined.

### Supplementary Information

**Additional file 1: Figure S1.** k-mer distribution of the *H. tibetana* genome. (a) k-mer = 17 Depth and k-mer number frequency distribution. (b) k-mer = 17 depth and k-mer type frequency distribution. **Figure S2.** The Venn diagram of gene function annotations in *H. tibetana* obtained using four databases, including InterPro, Swiss-Prot, NR, and KEGG. **Figure S3.** Gene family and KEGG enrichment analysis of unique genes in *H. tibetana*. (A) GO terms enriched in unique genes of *H. tibetana*. (B) KEGG pathways enriched in unique genes of *H. tibetana*. **Figure S4.** All differentially expressed genes (DEGs) between different tissues. **Figure S5.** All DEGs were classified into four clusters according to their expression patterns.

**Additional file 2: Table S1.** Statistics of *H. tibetana* assembled chromosomes. **Table S2.** Statistics of predicted protein-coding genes in* H.tibetana*. **Table S3.** Statistical information of non-coding RNA in *H.tibetana* genome. **Table S4.** Evaluation of genome assembly completeness with 1614 BUSCO groups. **Table S5.** Statistics of repetitive sequences in the *H. tibetana* genome. **Table S6.** Genes involved in the flavonoid pathway. **Table S7.** Expression level of genes in *H. tibetana*. **Table S8.** Primers used for qRT-PCR.

## Data Availability

The genome assembly of the *H. tibetana* is available on the NCBI website (NCBI) under accession number PRJNA1070417 [64]. The raw sequencing data used for de novo whole-genome assembly and transcriptome data of Illumina RNA-seq are available from the BioProject under accession number PRJNA1070417 [64].

## Abbreviations

QTP: Qinghai-Tibetan Plateau
Mya: Million years ago
PAL: Phenylalanine ammonia lyase
C4H: Cinnamic acid hydroxylase
4CL: 4-Coumaric acid CoA ligase
CHS: Chalcone synthase
CHI: Chalcone isomerase
FNSI and FNS II: Flavone synthase I and II
F3′5′H: Flavonol 3′5′-hy-droxylase
FLS: Flavonol synthase
DFR: Dihydroflavonol 4-reductase
TEs: Transposable elements
CEGMA: Core eukaryotic gene mapping approach
LTRs: Long-terminal repeats
TDC: Tryptophan decarboxylase
CIPK: CBL-interacting protein kinase
FAH2: Fatty acid 2-hydroxylase 1-like
DIR: Dirigent protein-like
WGD: Whole-genome duplication
POLD3: DNA-directed DNA polymerase
XRCC2: X-ray repair cross complementing 2
SV: Structural variation
APEX2: AP endonuclease 2 protein
POLD2: DNA polymerase delta subunit 2
CIPK: CBL-interacting protein kinase
ORFs: Pen reading frames
qRT-PCR: Quantitative real-time RT-PCR
DEGs: Differentially expressed genes
