# Supplementary material for: Chromosome-level genome assembly of Hippophae tibetana provides insights into high-altitude adaptation and flavonoid biosynthesis
Source: BMC Biol. 2024 Apr 12;22:82. doi: 10.1186/s12915-024-01875-4 (PMC11015584; doi:10.1186/s12915-024-01875-4)
Supplement: Supplementary file 1 — Additional file 1: Figure S1. k-mer distribution of the H. tibetana genome. (a) k-mer = 17 Depth and k-mer number frequency distribution. (b) k-mer = 17 depth and k-mer type frequency distribution. Figure S2. The Venn diagram of gene function annotations in H. tibetana obtained using four databases, including InterPro, Swiss-Prot, NR, and KEGG. Figure S3. Gene family and KEGG enrichment analysis of unique genes in H. tibetana. (A) GO terms enriched in unique genes of H. tibetana. (B) KEGG pathways enriched in unique genes of H. tibetana. Figure S4. All differentially expressed genes (DEGs) between different tissues. Figure S5. All DEGs were classified into four clusters according to their expression patterns. [file 12915_2024_1875_MOESM1_ESM.docx]

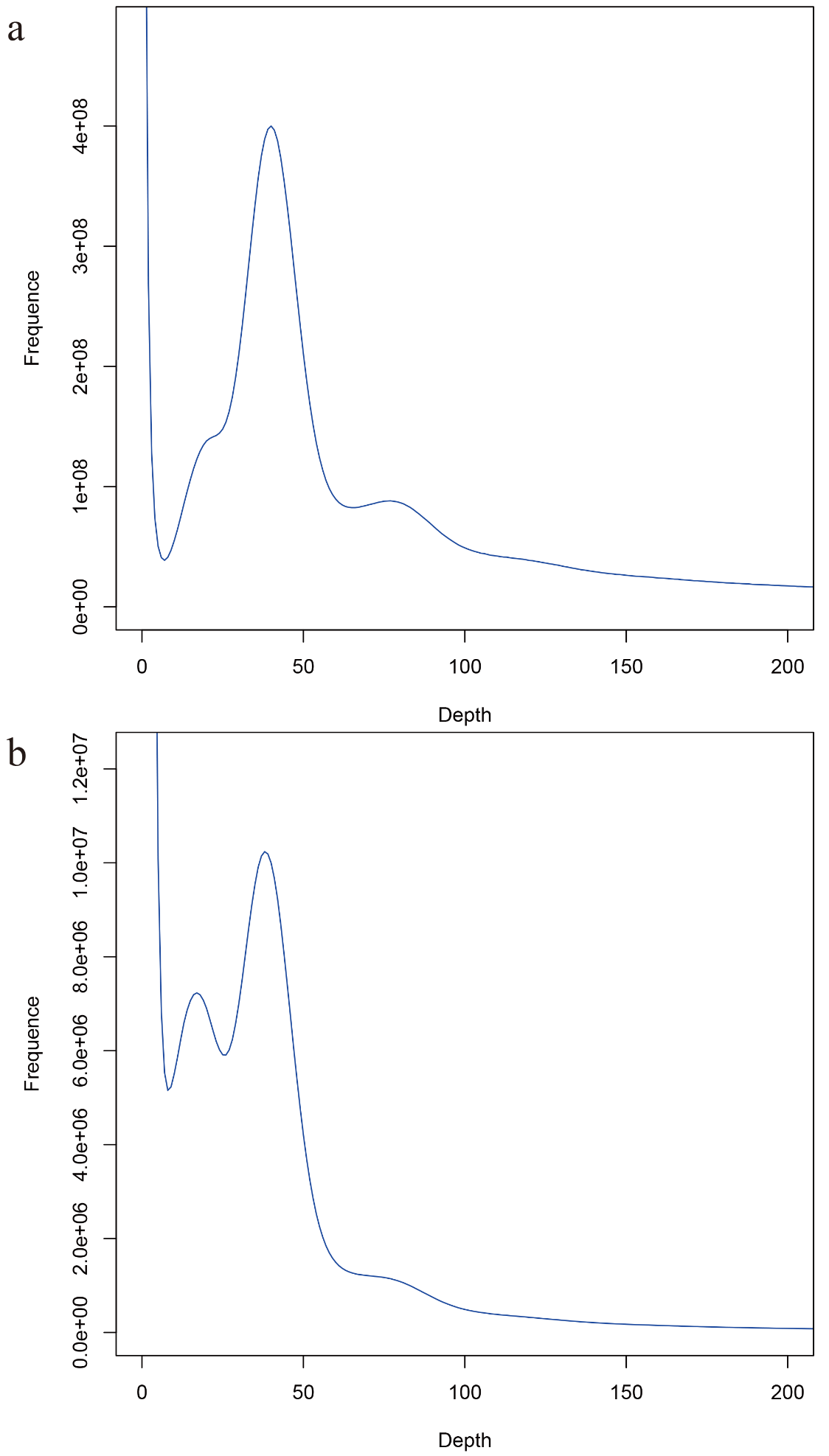


**Figure S1.** k-mer distribution of the *Hippophae tibetana* genome. (a) k-mer = 17 Depth and k-mer number frequency distribution. (b) k-mer = 17 depth and k-mer type frequency distribution.


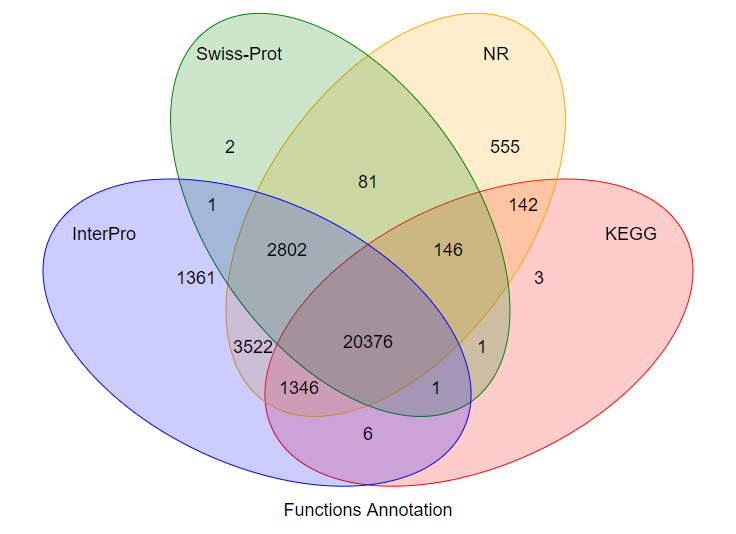


**Figure S2.** The Venn diagram of gene function annotations in *Hippophae tibetana* obtained using four databases, including InterPro, Swiss-Prot, NR, and KEGG.


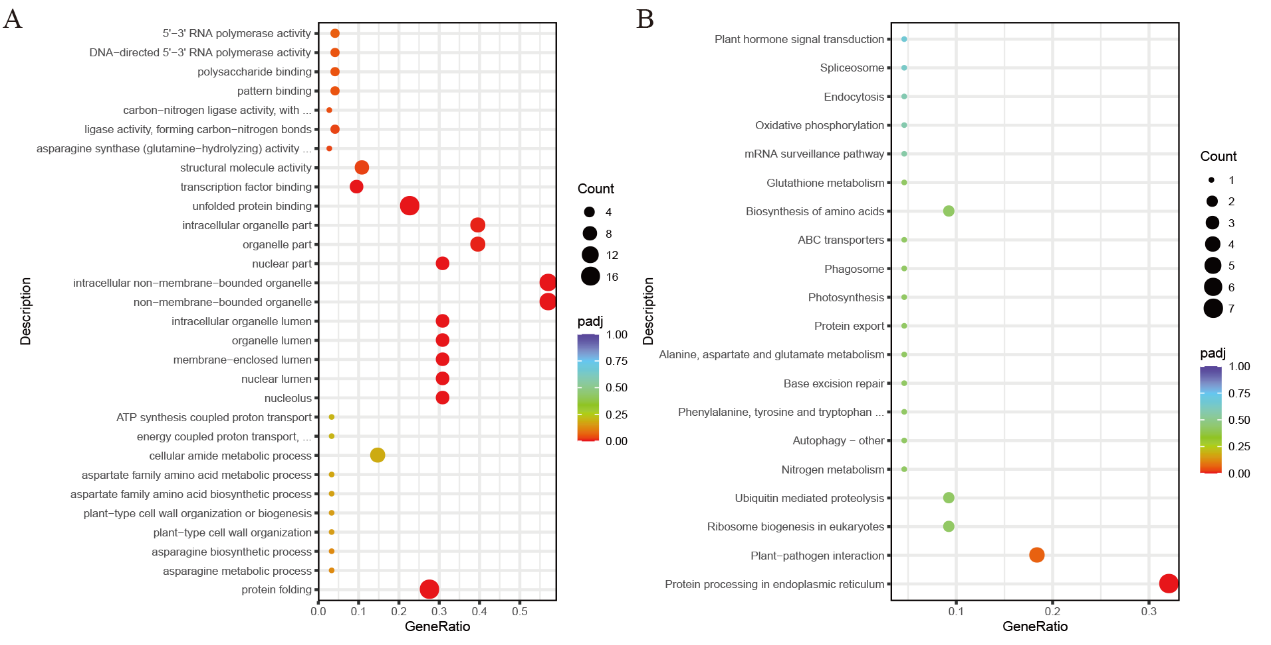


Figure S3**. Gene family and KEGG enrichment analysis of unique genes in *Hippophae tibetana*.** (A) GO terms enriched in unique genes of *Hippophae tibetana*. (B) KEGG pathways enriched in unique genes of *Hippophae tibetana*.


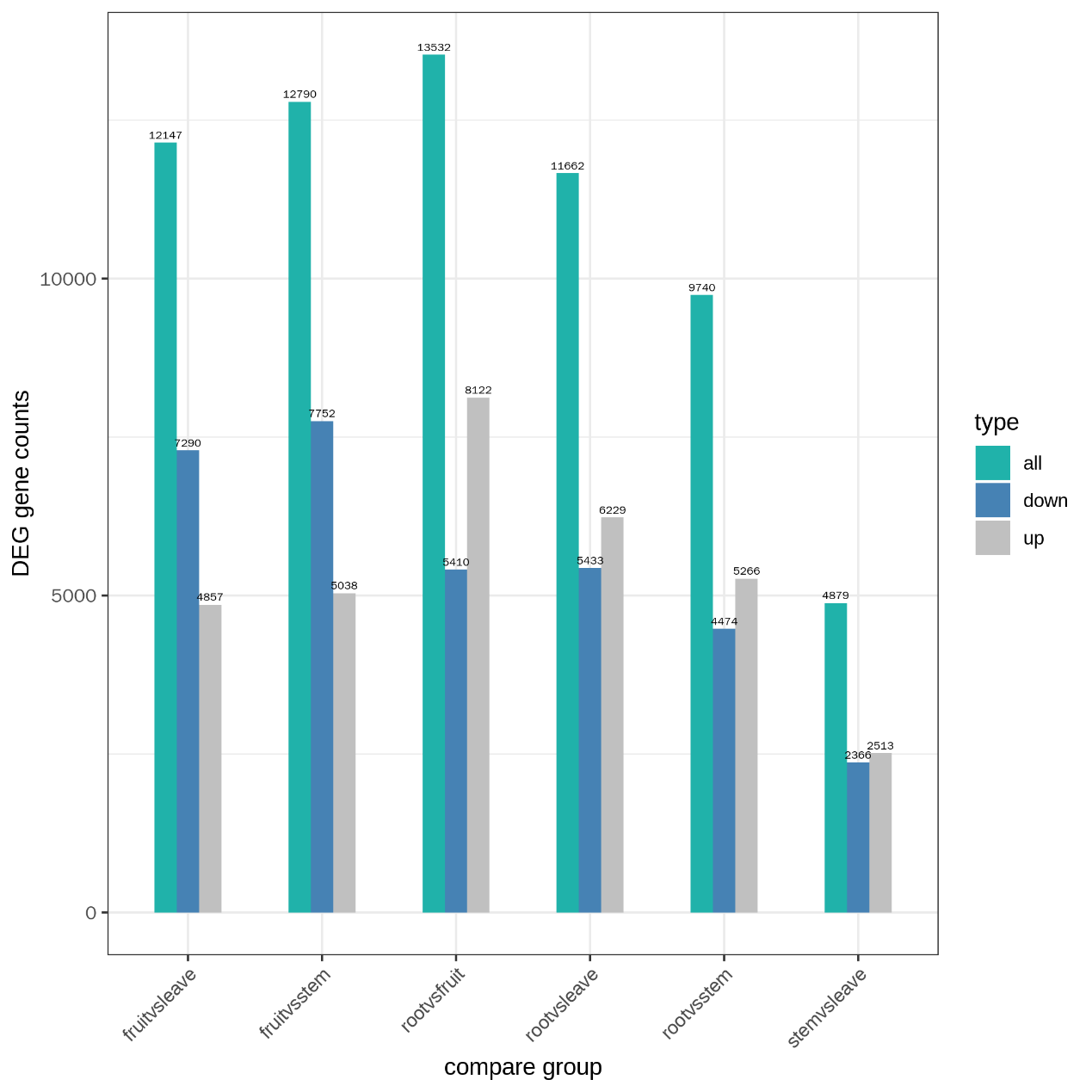
 **Figure S4.** All differentially expressed genes (DEGs) between different tissues.


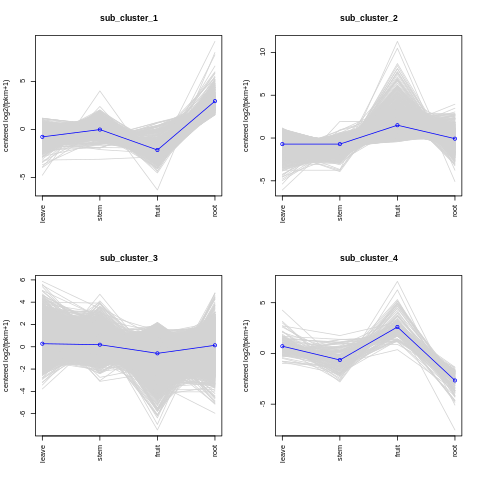


**Fig****ure S5.** All differentially expressed genes (DEGs) were classified into four clusters according to their expression patterns.
